# Blood cell transcriptomic-based early biomarkers of adverse programming effects of gestational calorie restriction and their reversibility by leptin supplementation

**DOI:** 10.1038/srep09088

**Published:** 2015-03-13

**Authors:** Jadwiga Konieczna, Juana Sánchez, Mariona Palou, Catalina Picó, Andreu Palou

**Affiliations:** 1 Laboratory of Molecular Biology, Nutrition and Biotechnology (Nutrigenomics), University of the Balearic Islands (UIB) and CIBER Fisiopatología de la Obesidad y Nutrición (CIBEROBN), Palma de Mallorca, Spain

## Abstract

The challenge of preventing major chronic diseases requires reliable, early biomarkers. Gestational mild undernutrition in rats is enough to program the offspring to develop later pathologies; the intake of leptin, a breastmilk component, during lactation may reverse these programming effects. We used these models to identify, in peripheral blood mononuclear cells (PBMCs), transcriptomic-based early biomarkers of programmed susceptibility to later disorders, and explored their response to neonatal leptin intake. Microarray analysis was performed in PBMCs from the offspring of control and 20% gestational calorie-restricted dams (CR), and CR-rats supplemented with physiological doses of leptin throughout lactation. Notably, leptin supplementation normalised 218 of the 224 mRNA-levels identified in PBMCs associated to undernutrition during pregnancy. These markers may be useful for early identification and subsequent monitoring of individuals who are at risk of later diseases and would specifically benefit from the intake of appropriate amounts of leptin during lactation.

Prevention is the new paradigm in personalized nutrition and medicine. This perspective may benefit the approach to major chronic diseases such as obesity and its related pathologies, because of their increased worldwide prevalence and difficulty to treat. The development of effective preventive strategies requires reliable early biomarkers. The biomarkers that are conventionally used to predict the risk of obesity and its co-morbidities mainly involve body weight, BMI, blood pressure, lipid profile, and plasma glucose and insulin levels. Bearing in mind the complexity of the mechanisms contributing to obesity and its related pathologies, there is a need for multiple novel biomarkers beyond the prediction of traditional metabolic risk factors^1^,^2^,^3^. Application of early transcript-based metabolic markers might be more beneficial, providing more information regarding disease or physiological changes occurring in the body before phenotipic features become evidente, as well as giving more time to intervene in the prevention of a future disease^4^,^5^. Studies on molecular mechanisms revealing effects of diet on health usually imply invasive tissue biopsies^6^. However, suitability of biomarkers for effective use requires that they can be easily accessed in humans. Peripheral blood mononuclear cells (PBMCs) provide an attractive alternative, as they can be easily and repeatedly collected in sufficient quantities^5^,^7^,^8^,^9^. These cells are able to respond to internal and external signals, and thereby have been proposed as a source of biomarkers of health and disease, as their gene expression profile may reflect the physiological and pathological state of the organism^8^,^9^,^10^,^11^,^12^.

Identification of early biomarkers requires the use of suitable animal models with a different predisposition to subsequent diseases. Of particular interest are the models whose bias is acquired due to the influence of common environmental and nutritional factors. In this sense, there is an accumulating body of evidence showing that maternal nutrition during the perinatal period has significant effects on fetal growth and can exert powerful influences upon long-term health and well-being^13^,^14^. In this context, human and animal models associate maternal calorie-restriction during pregnancy with adverse health outcomes in adult offspring, mainly related, but not restricted, to the development of obesity, cardiovascular diseases and type II diabetes, particularly when exposed postnatally to obesogenic conditions^15^,^16^,^17^,^18^. Although the factors involved in the programming effects are not clearly known, the role of the hormone leptin is receiving special attention^19^,^20^.

Leptin is mainly produced by the adipose tissue, but is also naturally present in significant amounts in breast milk^21^. In addition to the well established role of leptin in the regulation of energy homeostasis in adulthood^22^,^23^, it has been shown to play a critical role during development by programming hypothalamic circuit formation during a neonatal restricted window, which coincides with an increase in plasma leptin levels, the so called leptin surge^24^,^25^. In this sense, our group and others have evidenced that maternal undernourishment during gestation in rodents, which is associated to a disturbed neonatal leptin surge^15^,^26^, perturbs hypothalamic structure and function^27^,^28^,^29^, and hence results in a greater propensity for obesity and insulin resistance development in adulthood. More recently, we have also shown that daily administration of oral leptin at physiologic doses throughout lactation to the offspring of moderate calorie-restricted dams during gestation, was able to revert most of the developmental malprogramming effects on hypothalamic structure and function^30^. Similarly, daily subcutaneous injection of leptin into neonatal rats born to undernourished mothers has been shown to prevent the development of a programmed trend towards obesity and other metabolic alterations in later life^31^,^32^. Positive effects of oral leptin during lactation are not restricted to the offspring of calorie-restricted dams. We described for the first time that oral treatment with physiological doses of leptin throughout lactation to the offspring of *ad libitum* fed dams during gestation, protects againsts age-related overweight and prevents metabolic alterations associated to the intake of a high-fat diet^19^,^33^,^34^. Other studies – which addressed the effects of early postnatal diet on healthy, lasting outcomes – revealed that infants who are breastfed are less likely to become obese than those who are exclusively formula fed^35^,^36^, and a negative association has been found between maternal milk leptin levels and body weight increase of infants^37^, which has been confirmed by other groups^38^,^39^. Thus, leptin may be considered as an essential factor during lactation in the protection against subsequent overweight, obesity and its co-morbidities in later life, and may also be worth considering when searching for strategies to reverse programmed susceptibility to obesity acquired by fetal undernutrition^40^,^41^.

Therefore, in the present study, we used whole genome transcriptome profiling of PBMCs of pups from calorie-restricted dams during gestation in order to identify early biomarkers of programmed susceptibility to obesity-related chronic diseases and to determine whether supplementation with physiological doses of leptin during the suckling period is able to revert these markers of developmental malprogramming.

## Results

### Anthropometric measurements and circulating parameters in the offspring

Description of the phenotype of pups at birth and after weaning has been previously published^30^. Here we summarize data related to body weight, body fat content and blood parameters (Table 1). Although mild maternal calorie restriction during gestation had no effects on the body weight of neonate rats, after weaning CR pups weighed less and exhibited lower body fat content than controls (*p* < 0.05; LSD *post hoc* one-way ANOVA test). Mentioned parameters were not influenced by oral leptin treatment throughout lactation (*p* < 0.05; LSD *post hoc* one-way ANOVA test)^30^.

As previously published^30^, either maternal calorie restriction during gestation or leptin treatment throughout lactation did not affect circulating glucose and insulin levels at juvenile age (day 25); however, oral leptin treatment during the suckling period partially reverted the decreased plasma leptin levels found in CR males (*p* < 0.05; LSD *post hoc* one-way ANOVA test).

### Gene expression in PBMCs of male animals at the age of 25 days based on whole-genome microarray analysis

**Figure 1:**
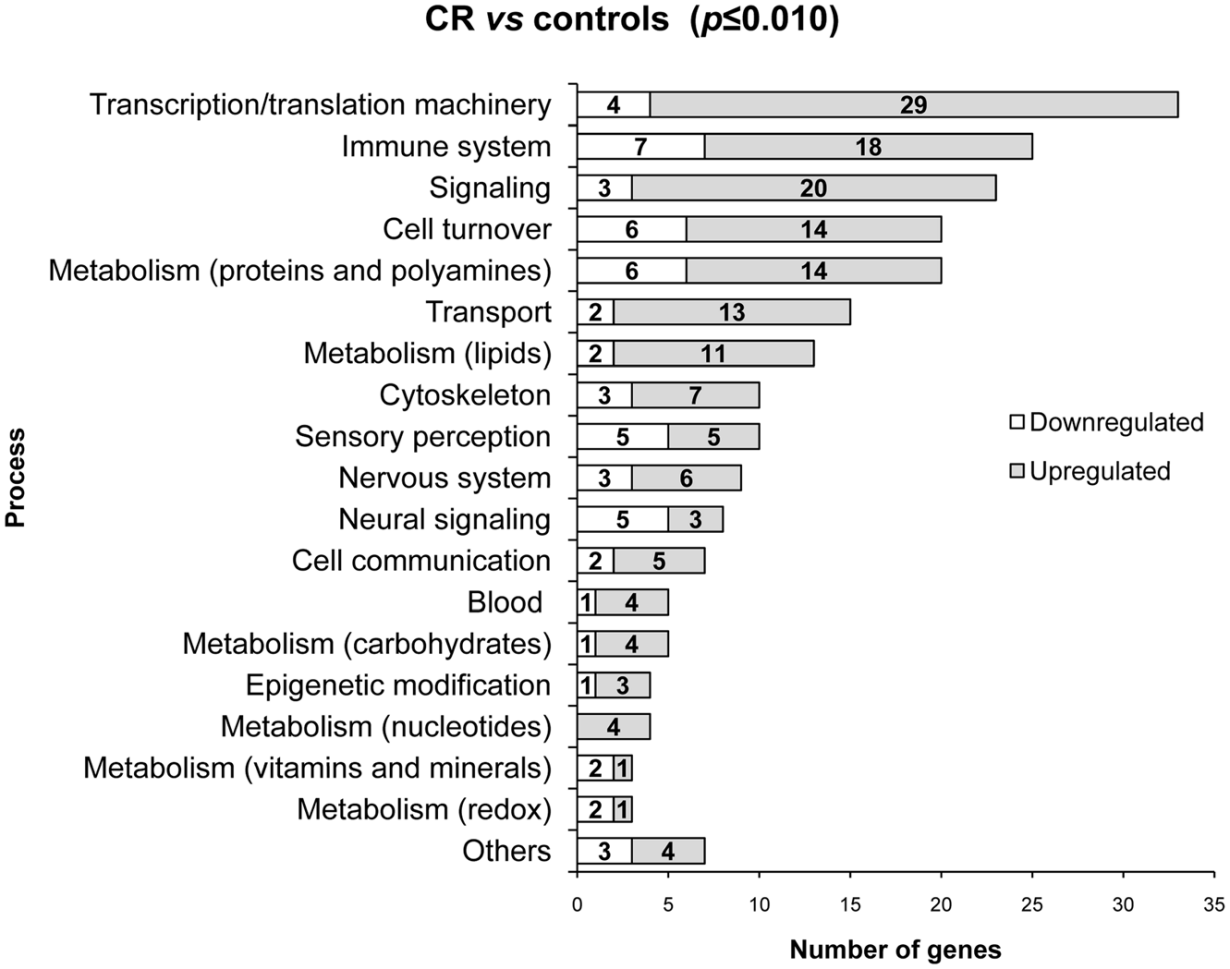
Microarray data classification into biological processes of the genes differentially expressed in PBMCs between male offspring of control and calorie-restricted dams during gestation (CR) at the age of 25 days. Threshold of significance was set at *p* ≤ 0.010 (limma *t*-test). The number of genes down- or up-regulated is indicated for each group of genes.

Whole genome microarray analysis was performed in PBMCs of 25 day-old male animals belonging to the 3 groups of animals (control, CR, and CR-Leptin). Of the 45,220 probes tested, 36,122 probes remained after background correction, normalization and merged replicated clones and were further taken into account. In all, 473 unique genes were found to be significantly different between control and CR male animals (*p* ≤ 0.010; limma *t*-test). Using available databases (Genecards, KEGG, NCBI, Reactome, UniProt, USCN, WikiPathways), these genes were classified into several biological processes according to their function. 249 genes were unknown and were not considered. From the remaining 224 known genes, 58 exhibited down-regulation and 166 up-regulation (Figure 1). The processes with the highest number of differentially expressed genes were related to transcription/translation machinery, immune system, signaling, cell turnover and metabolism of proteins and polyamines. Other processes with a notable number of genes whose expression was altered were related to transport, metabolism of lipids, cytoskeleton, sensory perception, nervous system and neural signaling. The remaining genes were related to cell communication, blood (including genes related to blood coagulation, or with specific functions of hemapoietic cells), metabolism of carbohydrates, epigenetic modification, metabolism of nucleotides, metabolism of vitamins and minerals, and redox metabolism. Other biological processes such as fertilization, detoxification, angiogenesis, and embryonic development, were grouped with the name ‘others'.

**Figure 2:**
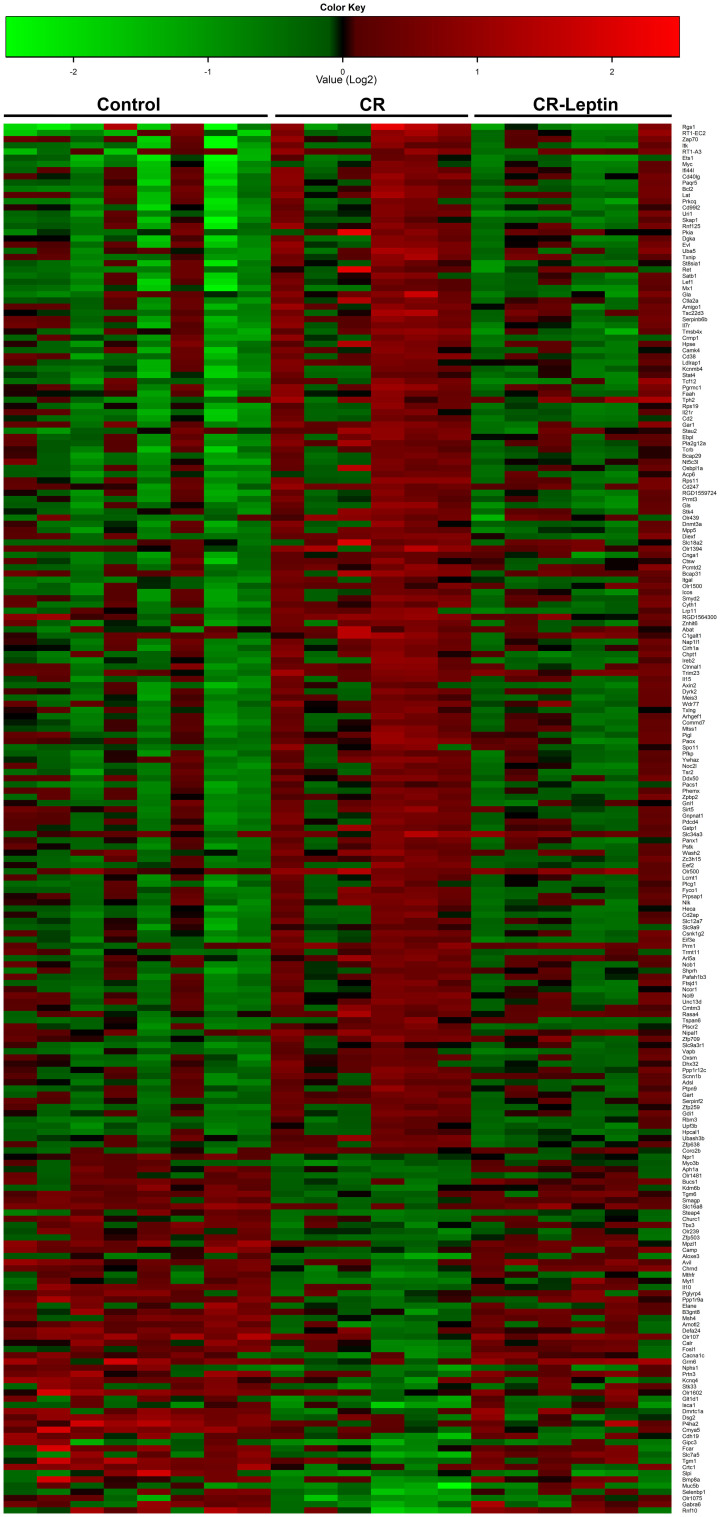
Heat map representing indicidual expression data of the genes differentially expressed in PBMCs between male offspring of control and calorie-restricted dams during gestation (CR) at the age of 25 days. Rows represent the 224 genes differentially expressed between control and CR pups (p < 0.010, limma *t*-test), sorted by fold change. Columns represent the log2 value of the expression of each gene in each animal studied.

Subsequently, we performed statistical analyses to compare mRNA expression levels between CR and CR-Leptin groups and between CR-Leptin and control groups. Of the 224 known genes differently expressed in CR pups *vs* controls (*p* ≤ 0.010; limma *t*-test), 22 of these were found to be different between CR and CR-Leptin groups (*p* ≤ 0.010; limma *t*-test) and simultaneously not statistically different between control and CR-Leptin pups; thus, we assumed that the expression levels of these genes were totally reverted by oral leptin treatment throughout lactation. The detailed list of these genes is shown in Table 2. Transcript levels of the remaining 196 genes, which appeared to be affected by moderate maternal calorie restriction during gestation, became partially reverted by leptin treatment throughout lactation (Supplementary Table 1). These include the set of genes whose expression profile differed significantly in CR *vs* control males (*p* ≤ 0.010; limma *t*-test) but did not differ significantly in CR-Leptin *vs* CR and CR-Leptin *vs* control animals. Only 6 of the 224 genes whose expression levels were changed due to gestational calorie restriction (CR *vs* control males, *p* ≤ 0.010; limma *t*-test) were not reverted by leptin treatment throughout lactation, as their expression levels did not differ between CR and CR-Leptin groups and were significantly different between control and CR-Leptin groups (*p* ≤ 0.010; limma *t* test). These genes were the following: cardiomyopathy associated 5 (*Cmya5*); defensin, alpha, 24 (*Defa24*); desmoglein 2 (*Dsg2*); secretory leukocyte peptidase inhibitor (*Slpi*); tryptophan hydroxylase *2 (Tph2)*; and tripartite motif-containing 23 (*Trim23*). These are related to the immune system, cell turnover, neural signaling and metabolism of protein processes. The results from the array concerning the 224 genes differently expressed between control and CR groups across individual samples are depicted in a heat map (Figure 2). This figure illustrates the similarity between control and CR-Leptin groups concerning the expression pattern of these genes.

### Confirmation of microarray results in male animals by RT-qPCR and verification in females

**Figure 3:**
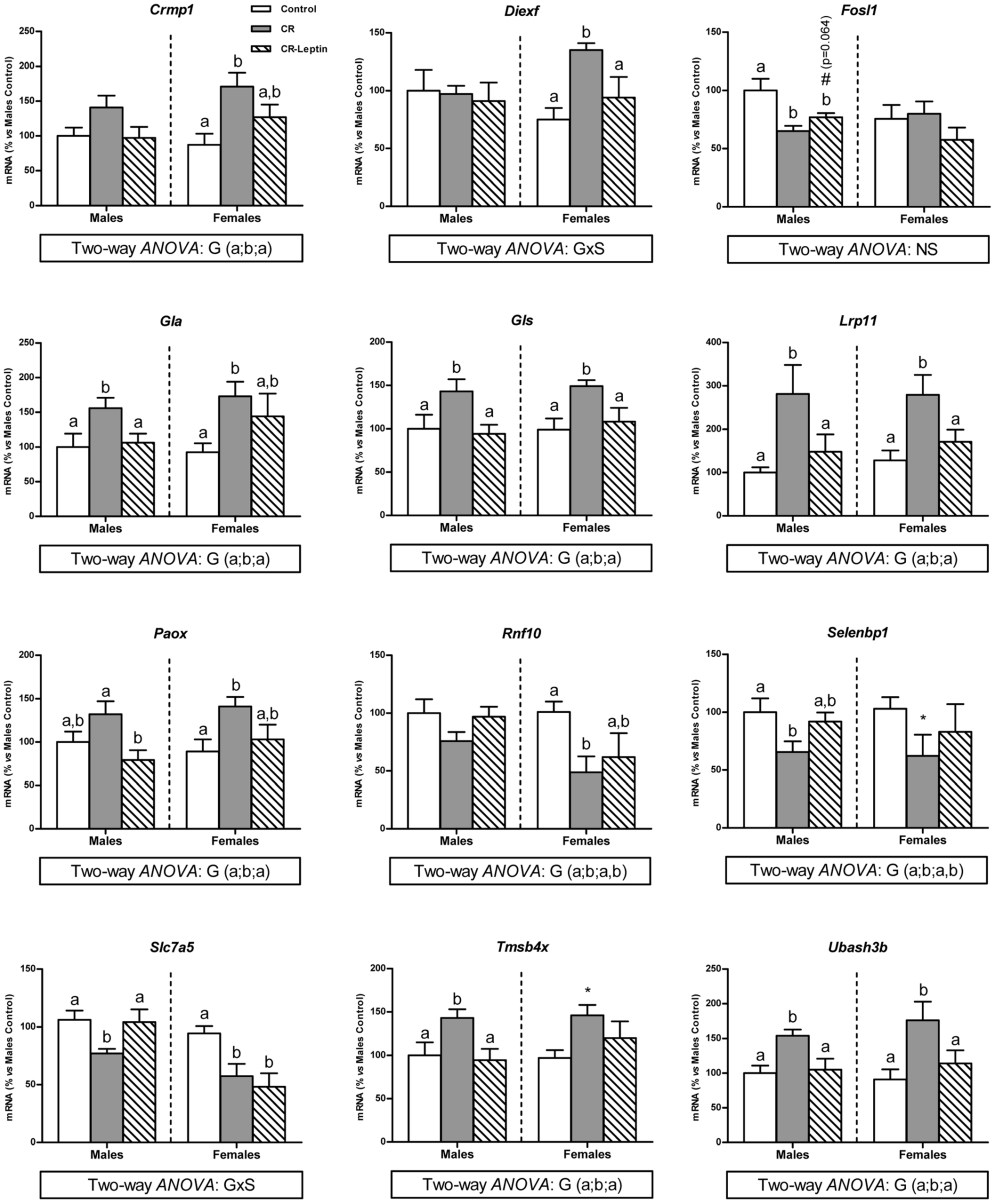
Q-PCR substantiation of microarray data of genes affected by gestational calorie restriction and totally reverted by oral leptin supplementation throughout lactation. To confirm and verify consistency of array findings, RT-qPCR was performed in PBMCs samples of both male and female offspring of control and calorie-restricted dams during gestation (CR) at the age of 25 days. mRNA levels were expressed as a percentage of the mean value of male controls. Data are mean ± S.E.M (n = 7–10 animals/group). Statistical analysis was performed by considering males and females as a whole (*p* ≤ 0.05; two-way ANOVA), and separately for each sex (*p* ≤ 0.05; one-way ANOVA). At any rate, data not sharing a common letter (a and b) are significantly different (a ≠ b) (*p* < 0.05; LSD *post hoc* one-way and two-way ANOVA). Symbols: G, effect of group; GxS; interaction between the effect of group and the effect of sex (*p* < 0.05; two-way ANOVA); *, CR *vs* Controls (*p* < 0.05; Student's *t* test); #, CR-Leptin *vs* CR (*p* < 0.05; Student's *t* test); NS, not statistically significant.

To confirm gene array findings in male pups as well as to verify whether the described changes were also occurring in females, expression levels of a selected set of genes − the 22 genes whose expression levels were completely normalized in leptin-treated CR male pups − were analysed by RT-qPCR. Among those genes, the expression levels were found to be quantifiable by RT-qPCR for: collapsin response mediator protein 1 (*Crmp1*); digestive organ expansion factor homolog (zebrafish) (*Diexf*); fos-like antigen 1 (*Fosl1*); galactosidase, alpha (*Gla*); glutaminase (*Gls*); low density lipoprotein receptor-related protein 11 (*Lrp11*); polyamine oxidase (exo-N4-amino) (*Paox*); ring finger protein 10 (*Rnf10*); selenium binding protein 1 (*Selenbp1*); solute carrier family 7 (amino acid transporter light chain, L system), member 5 (*Slc7a5*); thymosin beta 4, X-linked (*Tmsb4x*); and ubiquitin associated and SH3 domain containing, B (*Ubash3b*). The expression patterns of these genes in male and female animals are shown in Figure 3. RT-qPCR analysis fully confirmed most of the microarray data in male pups, and gene expression profile was largely similar in females. In detail, considering males and females, gestational calorie restriction resulted in higher mRNA levels of *Crmp1*, *Gla*, *Gls*, *Lrp11*, *Paox*, *Tmsb4x* and *Ubash3b*; in turn, leptin treatment throughout lactation significantly diminished transcript levels of these genes to the control values, reversing the effects observed in CR animals (*p* < 0.05; LSD *post hoc* two-way ANOVA test).

Differences concerning *Rnf10* and *Selenbp1* expression levels identified using the microarrays were partially confirmed by RT-qPCR analysis. Gestational calorie restriction brought about down-regulation of mRNA levels of both genes in CR animals (*p* ≤ 0.05; LSD *post hoc* two-way ANOVA). In turn, leptin supplementation throughout lactation partially normalized their levels to those observed in controls (*p* ≤ 0.05; LSD *post hoc* two-way ANOVA).

RT-qPCR analysis revealed that mRNA levels of *Diexf* and *Slc7a5* showed different expression pattern between sexes (interaction between group and sex; *p* ≤ 0.05; two-way ANOVA). Leptin treatment throughout lactation resulted in total reversion of increased *Diexf* mRNA expression levels in CR females (*p* ≤ 0.05; LSD *post hoc* one-way ANOVA), whereas no statistical differences were observed in males either due to gestational calorie restriction or due to leptin treatment throughout lactation. Regarding *Slc7a5* mRNA levels, as confirmed with both techniques used, decreased expression levels of this gene in CR animals were restored due to leptin treatment throughout lactation in males, but not in females (*p* ≤ 0.05; LSD *post hoc* one-way ANOVA).

RT-qPCR analysis of *Fosl1* did not fully confirm the expression pattern of this gene observed with the microarray analysis: although its expression levels were confirmed to be diminished in CR males *vs* controls (*p* ≤ 0.05; LSD *post hoc* one-way ANOVA), leptin treatment throughout lactation did not restore its control levels (*p* ≤ 0.05; LSD *post hoc* one-way ANOVA). Nevertheless, single comparison between groups revealed that CR-Leptin males exhibited a trend to higher *Fosl1* mRNA levels relative to their CR counterparts (*p* = 0.064; Student's *t* test). Expression levels of *Fosl1* in females were not different between groups.

## Discussion

One of the major challenges in developed societies is to implement therapeutic and, even more interestingly, preventive strategies, to fight the increasing prevalence of obesity and its related metabolic disorders. In this regard, there is an increased need for identification of reliable, early biomarkers of propensity to these alterations^1^. This could serve as a tool to improve the accuracy of disease diagnose and effectiveness of its prevention, as well as to monitor the efficacy of therapy used. Therefore, adding novel multimarkers beyond the traditional risk factors for obesity development may substantially improve early identification of susceptible individuals in order to implement effective therapeutic and/or preventive strategies. A growing number of studies exploring nutrigenomic-derived biomarkers focus on blood cells as a surrogate tissue^4^,^8^,^9^,^42^,^43^, since they can be easily and repeatedly collected in humans, and hence may give a chance to apply results obtained from animal studies into humans. On the other hand, regarding obesity prevention, neonatal leptin treatment in rats has been found to prevent age- and high fat diet feeding-related overweight and other metabolic alterations^19^,^33^,^34^. Moreover, the intake of adecuate amounts of leptin during the suckling period has been proposed as one of the strategies for reversing the effects of metabolic disorders induced as a consequence of developmental programming^30^. Therefore, in this study we undertook these issues and attempted to identify early biomarkers that predict programmed susceptibility to obesity-related chronic diseases caused by moderate undernutrition during gestation and to monitor the response to neonatal leptin treatment using transcript-based biomarkers identified in PBMCs.

The outcomes of maternal undernutrition during gestation on the offspring have been broadly discussed in scientific literature. This prenatal condition exerts adverse health effects on the offspring, leading to hyperphagia, hypertension and greater fat accumulation^16^,^18^, impaired insulin and leptin sensitivity^15^, alterations in the structure and expression of neuropeptides regulating feeding behaviour in hypothalamus^28^,^29^ as well as alterations in adipose tissue and stomach sympathetic innervations^44^,^45^. Our study, for the first time, reveals, at transcriptional level, the whole of the detrimental programming sequelae induced by mild gestational calorie restriction, through whole-genome transcriptome profiling of PBMCs of male offspring. The rational for performing microarray analysis only in males results from previous findings indicating that the overall detrimental effects of this prenatal condition were more marked in males than in females, particularly in terms of overweight and fat accumulation^15^,^16^,^46^. Herein, we found that prenatal food restriction affected mRNA levels of 224 genes expressed in PBMCs. Induced changes in gene expression are mainly related to transcription and translation machinery, followed by genes related to immune system, which is not surprising as PBMCs are a subset of white blood cells. Gestational calorie restriction also affected PBMCs expression of genes involved, among others, in signalling, cell turnover, metabolism, transport, cytoskeleton, sensory perception, nervous system and neural signaling. Hence, we identified a comprehensive set of easily assessed transcript-based biomarkers that reflect a wide system of biological processes and can improve prediction of the programmed susceptibility to obesity and other pathologies induced by fetal undernutrition. Although it is true that, nowadays, the phenomenon of prenatal maternal undernutrition does not seem to be responsible for the increasing prevalence of obesity in developed countries, it might account for the increasing trends in the incidence of obesity-related pathologies in developing countries where maternal malnutrition is still widespread^47^. Moreover, fetal undernutrition may also arise as a result of deficiency caused by maternal failure of complete absorption of food components^48^ and differences in seasonal food availability^47^.

Interestingly, we also show here the overall effects of leptin supplementation throughout the suckling period in reverting gene expression alterations in PBMCs induced by maternal undernutrition during gestation. Neonatal leptin treatment normalized the expression of almost all of the genes whose expression was affected by calorie restriction during gestation in male offspring, with the exception of 6 genes. Among the 218 genes whose expression was normalized, 196 genes reverted partially and 22 were totally reverted. The results highlight the potential role of leptin during the suckling period, which might be worth considering when searching for strategies to treat and/or prevent the programmed trend to obesity acquired by inadequate fetal nutrition, particularly in susceptible subgroups. Microarray findings in PBMC samples from male animals were substantiated by analyzing expression levels of a selection of genes by RT-qPCR. We focused on the 22 genes whose expression levels were completely normalized in leptin-treated CR male pups. mRNA expression levels of the same set of genes were also determined in PBMC samples of female animals to verify whether their behaviour was similar to that of males. Among the 22 genes analyzed, expression levels of several of these (*Crmp1*, *Gla*, *Gls*, *Lrp11*, *Paox*, *Tmsb4x* and *Ubash3b*) emerged as particularly promising to be used as biomarkers because qPCR analyses fully confirmed microarray data and showed similar behavior between males and females. In addition to the aforementioned genes, other genes such as *Rnf10*, *Selenbp1*, *Diexf* and *Slc7a5* may also be of interest. qPCR analysis of the expression of *Rnf10* and *Selenbp1* genes in both genders showed similar patterns to those found for males with the microarray analysis, although decreased expression levels occurring in CR animals *vs* their controls were only partially reverted by leptin treatment during lactation. Sexual dimorphism was found concerning the expression pattern of the *Diexf* and *Slc7a5* genes in blood cells as an effect of treatment. The increased expression levels of the *Diexf* gene became totally reverted by leptin treatment, but only in the group of females, and no changes were found in males. Concerning *Slc7a5*, decreased expression levels of this gene occurring in CR animals were only normalized by leptin treatment during lactation in males, but not in females. Details regarding the proteins encoded by these genes and their potential functions are included as supplementary material (Supplementary Table 2). Besides the annotated basal function of proteins encoded by these genes, some of them have been found to serve as a bridge for several key biological pathways, and showed a significant association with some of the disease outcomes, although their specific function in PBMCs has not been generally explored.

Herewith, we identified a comprehensive set of easily assessed transcript-based biomarkers, with a particular emphasis for 7 of them (*Crmp1, Gla, Gls, Lrp11, Paox, Tmsb4x and Ubash3b*), reflecting the reversion of a wide system of developmentally programmed biological processes induced by relative fetal undernutrition. Although the exact function of each of these genes in PBMCs remains to be determined, identification of these markers, indicators of efficacy of neonatal leptin treatment, supports evidence of the positive effects of leptin intake during the suckling period on later health programming.

Taken together, the results from this study show that leptin supplementation throughout lactation is able to revert the expression of most of the identified potential early biomarkers of programmed obesity risk and other metabolic alterations associated to undernutrition during pregnancy. These findings support the potential importance of leptin during lactation, a specific compound of maternal milk, which might be of relevance when considering strategies to treat and/or prevent the programmed trend to diseases acquired by inadequate fetal nutrition, particularly in susceptible subgroups. Moreover, the biomarkers identified in the present study, if validated in humans, may allow the identification and subsequent monitoring of individuals at early ages who are at greater risk of developing obesity and other pathologies, and whose alterations can be reverted by the intake of adequate amounts of leptin during lactation.

## Methods

### Ethics statement

All experiments were performed in accordance with the approved guidelines. The animal protocol was approved by the Bioethical Committee of the University of the Balearic Islands (Resolution Number 8453. June, 2010).

### Animals and Experimental Design

**Figure 4:**
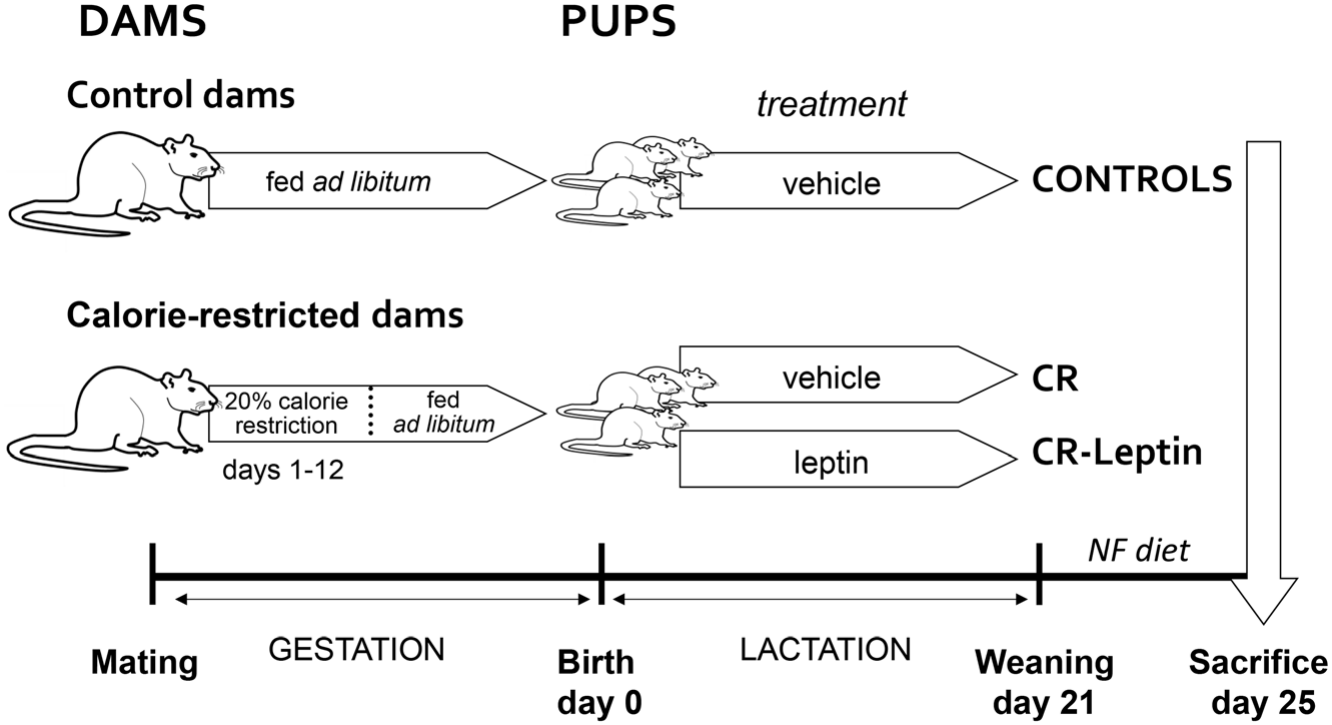
Schematic diagram of experimental design. After conception, the group of calorie-restricted dams was supplied with 20% calorie restriction diet from day 1 to day 12 of gestation, while the group of control dams was fed *ad libitum*. At birth, the offspring of control dams (control) was treated orally with vehicle throughout lactation, and the offspring of calorie restricted dams was divided into two groups: one group was also treated orally with vehicle (CR) and the other group was treated with a daily oral dose of leptin (CR-Leptin) throughout lactation. Daily oral dose of leptin was 5 times the average amount of daily leptin intake from maternal milk. Offspring was weaned at the age of 21 days and fed with normal fat diet until their sacrifice at the age of 25 days, when blood samples for plasma and PBMCs were obtained.

The animal protocol followed in this study has been previously described in detail^30^. Briefly, male and female Wistar rat pups coming from 17 litters and belonging to 3 different groups (control, CR and CR-Leptin) were used, according to the schema presented in Figure 4. Animals were housed under standard laboratory conditions (12 h dark and light cycle, 22°C) and allowed free access to water and a standard chow diet (3.3 kcal/g; Panlab, Barcelona, Spain), unless specified.

Virgin female rats were mated with male rats. After conception, the group of calorie-restricted dams (n = 10 animals) was submitted to 20% calorie restriction diet during the first 12 days of gestation, while the group of control dams (n = 7 animals) was fed *ad libitum*. After delivery, male and female offspring of calorie-resticted dams was distributed into two groups: CR and CR-Leptin. The group of CR-Leptin pups was treated orally throughout lactation with a water solution of recombinant murine leptin (PeproTech, London, UK). As established previously^19^, the dosis of leptin was at physiological level, progressively increased from 1 ng to 43.8 ng through this period. CR pups and the offspring of control dams (controls) were treated orally with the vehicle (water).

At weaning (day 21), pups from control, CR and CR-Leptin groups (35, 34 and 33 pups, respectively) were followed. They were housed (n = 2 per cage) and fed on a standard chow diet until their decapitation at the age of 25 days, under *ad libitum* feeding conditions. After sacrifice, trunk blood was collected from neck for PBMCs isolation and gene expression analysis (n = 10–11 animals per group), and for plasma circulating parameters determination (n = 6–8 per group). To obtain the plasma, blood was collected in heparinized tubes and centrifuged at 1000 *g* for 10 min. Animals used for analyses were from a minimum of six different litters.

### PBMC isolation

Trunk blood samples of control, CR and CR-Leptin rats collected at the age of 25 days under *ad libitum* feeding conditions, were used to isolate PBMCs. Peripheral blood samples were collected using EDTA (final concentration of 3–4 mM) as anticoagulant, and then diluted with an equal volume of buffered saline (isosmotic). PBMCs were immediately isolated by OptiPrep density-gradient separation (Sigma Aldrich Química SA, Madrid, Spain) according to the manufacturer's instructions.

### Total RNA isolation

Total RNA was extracted from PBMCs of control, CR and CR-Leptin male and female animals by EZNA® TOTAL RNA kit I (Omega Bio-Tek Inc., Norcross, GA, USA) following the manufacturer's instructions.

### Microarray processing

For microarray analysis, RNA from PBMC samples obtained from male offspring of controls, CR and CR-Leptin animals at the age of 25 days were used (n = 10–11/group). RNA samples were analyzed on Agilent 2100 Bioanalyzer with RNA 6000 Nano chips (Agilent Technologies, Barcelona, Spain). To ensure the high quality of RNA, only samples having a RIN number ≥ 8 were used for microarrays (n = 8/group). Then, 80 ng of RNA from each sample was reverse transcribed to complementary DNA (cDNA) using the Agilent Low Input Quick Amp Labeling kit (Agilent Technologies), according to the manufacturer's protocol. Half of the cDNA sample (10 μl) was used for the linear amplification of RNA and labeling with cyanine-3 (Cy3) or Cy5. For these reactions, half of the amounts indicated by the manufacturer were used^49^. Transcription and labeling were carried out at 40°C for 2 h. Then, the labeled and amplified cRNA samples were purified using Qiagen Rneasy MiniSpin columns (Qiagen, Madrid, Spain). The incorporation of dyes and cRNA concentration was measured using the NanoDrop ND 1000 spectrophotometer (NanoDrop Techonologies, Ins., Wilmington, DE). Of the 24 samples, 20 were used for microarray analysis. Four samples (2 samples in the CR and 2 samples in the CR-Leptin group) were excluded because of a low yield (<825 ng) and low specific activity (<8.0 pmol Cy3 or Cy5 per μg of cRNA). Subsequently, each sample containing 825 ng of cRNA labeled with Cy5 and 825 ng of Cy3 pool were hybridized on 4 × 44 K G2519F rat whole genome Agilent microarrays (Agilent Technologies) for 17 h at 65°C in hybridization chambers in an oven rotating at 10 rpm (Agilent Technologies). After hybridization, the arrays were washed using Gene Expression (GE) Wash Buffer Kit (Agilent Technologies). The arrays were rinsed in GE Wash Buffer 1 for 1 min, GE Wash Buffer 2 for 1 min, followed by acetonitrile for 10 s, and finally with a Stabilization and Drying Solution for 30 s at room temperature, according to the manufacturer's protocol (Agilent Technologies).

### Microarray data analysis

The arrays were scanned with an Agilent Microarray Scanner (Agilent Technologies). Scanned images were examined for visible defects and proper grid alignment. The intensities of the signals from each spot were quantified, and the raw data were extracted using Feature Extraction Software version 10.10.1.1 (Agilent Technologies). Background correction and normalization were performed within the Babelomics platform (http://www.babelomics.org), a suite of web tools for microarray data analysis^50^. Differential gene expression between groups of animals (CR *vs* controls, CR-Leptin *vs* CR, and CR-Leptin *vs* controls) was assessed using the limma package from Bioconductor, implemented into Babelomics web platform^51^. The threshold of significance for the test was set at *p* ≤ 0.010. Subsequently, a statistically generated list of genes was manually analyzed in regard to their biological information, obtained with the use of available databases (Genecards, KEGG, NCBI, Reactome, UniProt, USCN, WikiPathways) based on key biological domains, such as molecular function and biological process. Genes whose expression levels were significantly different between control and CR groups were plotted in a heatmap using R (v 3.1.1, R Development Core Team). To control for false positives we performed the Benjamin-Hochberg false discovery rate (FDR) test; all the genes with a p < 0.01 had an adjusted p value < 0.42. Fold change (FC), defined here as a difference in log2 mean values between experimental groups, was also calculated within the Babelomics platform.

### Real-time quantitative RT-polymerase chain reaction (RT-qPCR) analysis

To validate microarray data, mRNA expression levels of selected genes: collapsin response mediator protein 1 (*Crmp1*); digestive organ expansion factor homolog (zebrafish) (*Diexf*); fos-like antigen 1 (*Fosl1*); galactosidase, alpha (*Gla*); glutaminase (*Gls*); low density lipoprotein receptor-related protein 11 (*Lrp11*); polyamine oxidase (exo-N4-amino) (*Paox*); ring finger protein 10 (*Rnf10*); selenium binding protein 1 (*Selenbp1*); solute carrier family 7 (amino acid transporter light chain, L system), member 5 (*Slc7a5*); thymosin beta 4, X-linked (*Tmsb4x*); ubiquitin associated and SH3 domain containing, B (*Ubash3b*) were measured by RT-qPCR in PBMC RNA samples of control, CR and CR-Leptin male animals. In addition, mRNA expression levels of the same set of genes were analyzed in PBMC samples of control, CR and CR-Leptin female animals to verify the manner of their response to the treatments used.

For RT-qPCR analysis, 0.05 μg of PBMC total RNA was used for reverse transcription by using iScript™ cDNA synthesis kit (Bio-Rad Laboratories, S.A., Madrid, Spain) according to the manufacturer's protocol.

Real-time PCR was performed using the Applied Biosystems StepOnePlus™ Real-Time PCR Systems (Applied Biosystems, Madrid Spain) with Power SYBER Green PCR Master Mix (Applied Biosystems). Each PCR was performed from 1/5 dilution of the cDNA product and forward and reverse primers (5 μM each). The sequences of primers purchased from Sigma Genosys (Sigma Aldrich Química SA, Madrid, Spain) are described in Table 3. After an initial Taq activation at 95°C for 10 min, PCR was performed using 40 two-temperature cycles with the following cycling conditions: 95°C for 15 s and 60°C for 1 min. The threshold cycle (Ct) values were calculated by the StepOne Software v2.2.2, and the relative mRNA expression of each gene was expressed as a percentage of male control rats, using the 2^−ΔΔCt^ method^52^. Proteasome (prosome, macropain) subunit, alpha type 6 (*Psm6*) was chosen as reference gene because our microarray data showed equal expression of this gene across all groups.

### Statistical analysis

Data are expressed as mean ± SEM. The statistical analysis of microarray data has been described in detail in the previous section (microarray data analysis). Statistical significances were assessed by one-way (separately for each sex) and two-way ANOVA, and least significance difference (LSD) *post hoc* test. Student's *t* test was used to compare individual means. *P* < 0.05 was defined as the threshold of significance, unless stated. Statistics were computed with SPSS Statistics 19.0 (SPSS, Chicago, IL).

## Author Contributions

Conceived and designed the experiments: A.P. and C.P. Performed the experiment: J.K., J.S. and M.P. Analyzed the data: J.K., J.S. and C.P. Contributed reagents/materials/analysis tools: J.K., J.S. and M.P. Wrote the manuscript: J.K., C.P. and A.P. All authors revieved the final manuscript.

## Supplementary Material

Supplementary Information Suplementary Information

## Figures and Tables

**Table 1:** Offspring parameters: anthropometric measurements and circulating parameters in the offspring of rats with free access to standard chow diet (control), the offspring of 20% calorie restricted dams during the first 12 days of pregnancy (CR), and CR rats daily supplemented with physiological doses of leptin throughout lactation (CR-Leptin)

		Males			Females	
	Control	CR	CR-Leptin	Control	CR	CR-Leptin
**Anthropometric measurements**						
Body weight, day 1 (g)	6.57 ± 0.18	6.37 ± 0.11	6.45 ± 0.12	6.38 ± 0.08	6.15 ± 0.13	6.18 ± 0.12
Body weight, day 21 (g)	47.0 ± 0.9 **a**	43.2 ± 0.9 **b**	43.9 ± 0.9 **b**	45.4 ± 0.6 **a**	42.2 ± 0.8 **b**	42.8 ± 0.8 **b**
Body weight, day 25 (g)	65.5 ± 1.1 **a**	59.6 ± 1.2 **b**	60.2 ± 1.2 **b**	61.7 ± 0.7 **a**	58.5 ± 1.2 **b**	57.0 ± 1.0 **b**
Body fat, day 25 (%)	12.4 ± 0.3 **a**	10.1 ± 0.2 **b**	10.5 ± 0.2 **b**	12.2 ± 0.2 **a**	10.5 ± 0.2 **b**	10.5 ± 0.2 **b**
**Circulating parameters (day 25)**						
Glucose (mg/dL)	148 ± 4	145 ± 2	138 ± 4	149 ± 4	148 ± 5	142 ± 4
Insulin (ng/L)	170 ± 24	204 ± 33	221 ± 59	248 ± 41	240 ± 49	163 ± 52
Leptin (ng/L)	2206 ± 170 **a**	1798 ± 121 **b**	2042 ± 45 **a,b**	1689 ± 241	1461 ± 145	1414 ± 129

Body weight was measured on postnatal days 1, 21 and 25. The other parameters were determined on day 25. Data are mean ± S.E.M. For body weight at different days and body fat content, n = 16–17; for circulating parameters, n = 6–8. Each group is made up of animals coming from at least six different litters. Statistics: data not sharing a common letter are significantly different (a ≠ b) (p < 0.05; LSD *post hoc* one-way ANOVA test).

**Table 2:** Detailed list of genes whose expression in PBMC samples of male rats at the age of 25 days was affected by gestational calorie restriction and totally reverted by oral leptin supplementation throughout lactation

				CR *vs* Controls		CR-Leptin *vs* CR	
Biological process	Gene name	Gene symbol	Sequence ID	*P*	FC	*P*	FC
Cell communication	Small cell adhesion glycoprotein	Smagp	NM_182817	0.000	−0.50	0.006	+0.41
Cell turnover	Digestive organ expansion factor homolog (zebrafish)	Diexf	NM_001013986	0.002	+0.73	0.006	−0.66
	Fos-like antigen 1	Fosl1	NM_012953	0.003	−0.64	0.009	+0.60
	Selenium binding protein 1	Selenbp1	NM_080892	0.001	−0.93	0.007	+0.81
Cytoskeleton	Myosin IIIB	Myo3b	NM_001191901	0.007	−0.42	0.010	+0.43
	Thymosin beta 4, X-linked	Tmsb4x	NM_031136	0.010	+0.84	0.010	−0.91
Metabolism (carbohydrates)	Galactosidase, alpha	Gla	NM_001108820	0.004	+0.88	0.004	−0.96
Metabolism (lipids)	Arachidonate lipoxygenase 3	Aloxe3	NM_001105793	0.009	−0.54	0.005	+0.63
	Butyryl Coenzyme A synthetase 1	Bucs1	NM_001108502	0.004	−0.47	0.004	+0.51
	Low density lipoprotein receptor-related protein 11	Lrp11	NM_001106217	0.002	+0.70	0.001	−0.77
Metabolism (proteins and polyamines)	Glutaminase	Gls	NM_001109968	0.008	+0.74	0.007	−0.80
	Polyamine oxidase (exo-N4-amino)	Paox	NM_001106311	0.008	+0.62	0.010	−0.64
	Ubiquitin associated and SH3 domain containing, B	Ubash3b	NM_001191792	0.010	+0.45	0.000	−0.65
Neural signaling	Cholinergic receptor, nicotinic, delta	Chrnd	NM_019298	0.006	−0.56	0.004	+0.62
	Gamma-aminobutyric acid (GABA) A receptor, alpha 6	Gabra6	NM_021841	0.002	−1.00	0.004	+0.96
	Glutamate receptor, metabotropic 6	Grm6	NM_022920	0.010	−0.66	0.004	+0.79
Nervous system	Collapsin response mediator protein 1	Crmp1	NM_012932	0.004	+0.82	0.008	−0.78
	Myelin transcription factor 1	Myt1	NM_001108615	0.007	−0.57	0.005	+0.64
	Ring finger protein 10	Rnf10	NM_001011904	0.003	−1.22	0.010	+1.11
Signaling	Calcium channel, voltage-dependent, L type, alpha 1C subunit	Cacna1c	ENSRNOT00000009343	0.006	−0.65	0.009	+0.66
Transport	Fyve and coiled-coil domain containing 1	Fyco1	NM_001106870	0.003	+0.57	0.010	−0.51
	Solute carrier family 7 (amino acid transporter light chain), member 5	Slc7a5	NM_017353	0.007	−0.87	0.000	+1.20

Controls: the offspring of rats with free access to standard chow diet; CR: the offspring of 20% calorie restricted dams during the first 12 days of pregnancy; CR-Leptin: CR rats daily supplemented with physiological doses of leptin throughout lactation. *p-*values (*P*) of microarray data (limma *t*-test) and fold change (FC) values (calculated as the difference between Log2 means) of CR *vs* Controls and CR-Leptin *vs* CR comparisons are indicated; +, indicates upregulation; −, downregulation. Threshold of significance was set at *p* ≤ 0.010. Expression levels of these genes were not significantly different between control and CR-Leptin groups.

**Table 3:** Sequences of primers and amplicon size used for qRT-PCR

Gene	Forward primer (5′ to 3′)	Reverse primer (5′ to 3′)	Amplicon (bp)
*Crmp1*	TGATTGTTCCTGGTGGAGTG	GGATTTGGTGTCTGCTGCTT	248
*Diexf*	TTCTACGACAGGGTTTCCAAG	GCCATCTTCACCATTCATTTC	258
*Fosl1*	GCAGAAACCGAAGAAAGGAA	CTGGAGAAAGGGAGATACAAGG	261
*Gla*	CCCGAGAGGGATTCAAAG	TACCCCAGTCAGCAAATGTC	199
*Gls*	GGAGGGAAGGTTGCTGATTA	AGGACTGAAGACAAAAGGGAAC	133
*Lrp11*	ACAGACGACCACGCCATT	CCTGGGAAGCACAGTCACA	198
*Paox*	TGGCTGTCCTGAATACCTTCTT	TCAAAAACCATCACCTCCTTG	190
*Psm6*	TGGCTATGAGATTCCTGTGG	CTGTCTGCTTCACTCCTGCT	206
*Rnf10*	GGGGGAAAAGAAACAAGTGG	AAGGTGTCAGGGTCAGCAAA	124
*Selenbp1*	TGACCGCTTCCTCTACTTCA	CTCGTTTTCCCTTGACCACT	194
*Slc7a5*	CCTGCCTCTGCGTGCTACT	CCCTTGTCCTATGTCCTTTCC	155
*Tmsb4x*	GCTCCTTCCAGCAACCAT	GGGGCAGCACAGTCATTT	278
*Ubash3b*	ACTTCATCGGGCTCTTTGTG	TGTTCTGGGCGAGTTTCTCT	189

Abbreviations: Collapsin response mediator protein 1 *(Crmp1)*; Digestive organ expansion factor homolog (zebrafish) *(Diexf)*; Fos-like antigen 1 *(Fosl1)*; Galactosidase, alpha *(Gla)*; Glutaminase *(Gls)*; Low density lipoprotein receptor-related protein 11 *(Lrp11)*; Polyamine oxidase (exo-N4-amino) *(Paox)*; Proteasome (prosome, macropain) subunit, alpha type 6 (*Psm6*); Ring finger protein 10 *(Rnf10)*; Selenium binding protein 1 *(Selenbp1)*; Solute carrier family 7 (amino acid transporter light chain, L system), member 5 *(Slc7a5)*; Thymosin beta 4, X-linked *(Tmsb4x)*; Ubiquitin associated and SH3 domain containing, B *(Ubash3b)*.
