# Supplementary material for: Blood cell transcriptomic-based early biomarkers of adverse programming effects of gestational calorie restriction and their reversibility by leptin supplementation
Source: Sci Rep. 2015 Mar 13;5:9088. doi: 10.1038/srep09088 (PMC4357898; doi:10.1038/srep09088)
Supplement: Supplementary Information — Suplementary Information [file srep09088-s1.pdf]

**Blood cell transcriptomic-based early biomarkers of adverse programming effects of gestational calorie restriction and their reversibility by leptin supplementation**

Jadwiga Konieczna, Juana Sánchez, Mariona Palou, Catalina Picó*, Andreu Palou

Supplementary table 1. Detailed list of genes whose expression levels in PBMC samples of male rats at the age of 25 days were affected by gestational calorie restriction and partially reverted by oral leptin supplementation throughout lactation.

| Biological Process | Gene Name | Gene Symbol | Sequence ID | CR vs Controls | | CR-Leptin vs CR | | CR-Leptin vs Controls | |
|---|---|---|---|---|---|---|---|---|---|
| | | | | P | FC | P | FC | P | FC |
| Blood | elastase, neutrophil expressed | Elane | NM_001106767 | 0.010 | -0.60 | 0.092 | +0.40 | 0.355 | -0.20 |
| | pan hematopoietic expression | Phemx | XM_002725757 | 0.010 | +0.60 | 0.057 | -0.46 | 0.501 | +0.15 |
| | phospholipid scramblase 2 | Plscr2 | NM_001014094 | 0.009 | +0.53 | 0.016 | -0.52 | 0.941 | +0.01 |
| | protein tyrosine phosphatase, non-receptor type 9 | Ptpn9 | NM_001013040 | 0.009 | +0.48 | 0.037 | -0.40 | 0.639 | +0.08 |
| | serpin peptidase inhibitor, clade F (alpha-2 antiplasmin, pigment epithelium derived factor), member 2 | Serpinf2 | NM_001011892 | 0.003 | +0.48 | 0.032 | -0.35 | 0.360 | +0.13 |
| Cell communication | CD99 molecule-like 2 | Cd99l2 | NM_134459 | 0.005 | +0.95 | 0.170 | -0.47 | 0.132 | +0.48 |
| | cadherin 19, type 2 | Cdh19 | NM_001009448 | 0.009 | -0.82 | 0.154 | +0.45 | 0.204 | -0.37 |
| | CKLF-like MARVEL transmembrane domain containing 3 | Cmtm3 | NM_001106164 | 0.007 | +0.53 | 0.133 | -0.30 | 0.206 | +0.23 |
| | catenin (cadherin associated protein), alpha-like 1 | Ctnnal1 | NM_001106649 | 0.008 | +0.66 | 0.062 | -0.47 | 0.426 | +0.18 |
| | integrin, alpha L | Itgal | NM_001033998 | 0.000 | +0.71 | 0.033 | -0.40 | 0.067 | +0.31 |
| | Pannexin 1 | Panx1 | NM_199397 | 0.006 | +0.59 | 0.013 | -0.55 | 0.855 | +0.04 |
| Cell turnover | B-cell CLL/lymphoma 2 | Bcl2 | NM_016993 | 0.002 | +0.96 | 0.031 | -0.69 | 0.351 | +0.27 |
| | bone morphogenetic protein 8a | Bmp8a | NM_001109432 | 0.007 | -0.90 | 0.049 | +0.67 | 0.464 | -0.23 |
| | dual-specificity tyrosine-(Y)-phosphorylation regulated kinase 2 | Dyrk2 | NM_001108100 | 0.008 | +0.65 | 0.158 | -0.35 | 0.195 | +0.30 |
| | v-ets erythroblastosis virus E26 oncogene homolog 1 (avian) | Ets1 | L20681 | 0.006 | +1.05 | 0.154 | -0.55 | 0.163 | +0.50 |
| | GIPC PDZ domain containing family, member 3 | Gipc3 | NM_001109282 | 0.008 | -0.83 | 0.076 | +0.57 | 0.355 | -0.27 |
| | headcase homolog (Drosophila) | Heca | NM_001107514 | 0.010 | +0.57 | 0.094 | -0.38 | 0.352 | +0.19 |
| | lymphoid enhancer binding factor 1 | Lef1 | NM_130429 | 0.007 | +0.89 | 0.223 | -0.40 | 0.114 | +0.49 |
| | mutS homolog 4 (E. coli) | Msh4 | NM_001106477 | 0.002 | -0.61 | 0.075 | +0.35 | 0.141 | -0.26 |
| | metastasis suppressor 1 | Mtss1 | NM_001130563 | 0.004 | +0.62 | 0.073 | -0.39 | 0.238 | +0.23 |
| | myelocytomatosis oncogene | Myc | NM_012603 | 0.002 | +1.04 | 0.173 | -0.43 | 0.048 | +0.61 |
| | nucleosome assembly protein 1-like 1 | Nap1l1 | NM_053561 | 0.005 | +0.68 | 0.022 | -0.57 | 0.628 | +0.11 |
| | programmed cell death 4 | Pdcd4 | NM_022265 | 0.007 | +0.59 | 0.084 | -0.39 | 0.309 | +0.21 |
| | protamine 1 | Prm1 | NM_001002850 | 0.000 | +0.56 | 0.241 | -0.18 | 0.017 | +0.37 |
| | SNF2 histone linker PHD RING helicase | Shprh | NM_001107470 | 0.008 | +0.55 | 0.292 | -0.22 | 0.096 | +0.33 |
| | taxilin gamma | Txlng | ENSRNOT00000006843 | 0.004 | +0.63 | 0.015 | -0.56 | 0.722 | +0.07 |
| | zinc finger protein 259 | Zfp259 | NM_001137646 | 0.007 | +0.47 | 0.125 | -0.27 | 0.220 | +0.20 |

| | | | | | | | | | |
|---|---|---|---|---|---|---|---|---|---|
| Cytoskeleton | advillin | Avil | NM_024401 | 0.005 | -0.55 | 0.029 | +0.45 | 0.565 | -0.10 |
| | CD2-associated protein | Cd2ap | NM_181475 | 0.008 | +0.57 | 0.034 | -0.47 | 0.639 | +0.09 |
| | coronin, actin binding protein, 2B | Coro2b | ENSRNOT00000020951 | 0.007 | +0.42 | 0.344 | -0.15 | 0.065 | +0.28 |
| | Enah/Vasp-like | Evl | NM_024147 | 0.009 | +0.92 | 0.083 | -0.62 | 0.355 | +0.30 |
| | protein phosphatase 1, regulatory subunit 12C | Ppp1r12c | NM_001191946 | 0.004 | +0.49 | 0.039 | -0.36 | 0.400 | +0.13 |
| | solute carrier family 9 (sodium/hydrogen exchanger), member 3 regulator 1 | Slc9a3r1 | NM_021594 | 0.003 | +0.51 | 0.335 | -0.16 | 0.032 | +0.35 |
| | serine/threonine kinase 33 | Stk33 | ENSRNOT00000019597 | 0.008 | -0.69 | 0.090 | +0.45 | 0.322 | -0.24 |
| | WAS protein family homolog 2 | Wash2 | NM_001127390 | 0.003 | +0.59 | 0.033 | -0.43 | 0.399 | +0.15 |
| Epigenetic modification | DNA (cytosine-5-)-methyltransferase 3 alpha | Dnmt3a | NM_001003958 | 0.008 | +0.73 | 0.076 | -0.50 | 0.366 | +0.23 |
| | lysine (K)-specific demethylase 6B | Kdm6b | NM_001108829 | 0.009 | -0.48 | 0.055 | +0.37 | 0.502 | -0.12 |
| | sirtuin 5 | Sirt5 | NM_001004256 | 0.010 | +0.60 | 0.060 | -0.45 | 0.496 | +0.15 |
| | SET and MYND domain containing 2 | Smyd2 | NM_206851 | 0.008 | +0.70 | 0.156 | -0.38 | 0.191 | +0.32 |
| Immune system | B-cell receptor-associated protein 31 | Bcap31 | NM_001004224 | 0.003 | +0.71 | 0.024 | -0.56 | 0.487 | +0.15 |
| | cathelicidin antimicrobial peptide | Camp | CB577971 | 0.009 | -0.54 | 0.121 | +0.33 | 0.273 | -0.21 |
| | Cd2 molecule | Cd2 | NM_012830 | 0.002 | +0.78 | 0.062 | -0.48 | 0.202 | +0.30 |
| | Cd247 molecule | Cd247 | NM_170789 | 0.003 | +0.75 | 0.160 | -0.35 | 0.096 | +0.40 |
| | CD40 ligand | Cd40lg | NM_053353 | 0.003 | +0.99 | 0.072 | -0.60 | 0.196 | +0.39 |
| | cytotoxic T lymphocyte-associated protein 2 alpha | Ctla2a | NM_001109115 | 0.001 | +0.88 | 0.026 | -0.60 | 0.243 | +0.28 |
| | IgA Fc receptor | Fcar | NM_201992 | 0.006 | -0.84 | 0.200 | +0.39 | 0.110 | -0.45 |
| | guanine nucleotide binding protein-like 1 | Gnl1 | NM_212500 | 0.006 | +0.60 | 0.034 | -0.47 | 0.529 | +0.13 |
| | inducible T-cell co-stimulator | Icos | NM_022610 | 0.007 | +0.70 | 0.054 | -0.52 | 0.435 | +0.19 |
| | interferon-induced protein 44-like | Ifi44l | XM_227820 | 0.005 | +1.01 | 0.089 | -0.61 | 0.223 | +0.40 |
| | interleukin 10 | Il10 | NM_012854 | 0.002 | -0.59 | 0.224 | +0.23 | 0.042 | -0.36 |
| | interleukin 15 | Il15 | NM_013129 | 0.006 | +0.65 | 0.135 | -0.35 | 0.171 | +0.30 |
| | IL2-inducible T-cell kinase | Itk | NM_001108825 | 0.009 | +1.07 | 0.213 | -0.51 | 0.143 | +0.56 |
| | linker for activation of T cells | Lat | NM_030853 | 0.005 | +0.96 | 0.078 | -0.61 | 0.266 | +0.35 |
| | myxovirus (influenza virus) resistance 1 | Mx1 | NM_173096 | 0.010 | +0.89 | 0.227 | -0.42 | 0.146 | +0.47 |
| | peptidoglycan recognition protein 4 | Pglyrp4 | NM_001191708 | 0.008 | -0.60 | 0.082 | +0.40 | 0.354 | -0.19 |
| | RT1 class I, locus A3 | RT1-A3 | NM_001008830 | 0.004 | +1.06 | 0.176 | -0.49 | 0.093 | +0.57 |
| | RT1 class Ib, locus EC2 | RT1-EC2 | M10094 | 0.004 | +1.22 | 0.183 | -0.55 | 0.088 | +0.67 |
| | src kinase associated phosphoprotein 1 | Skap1 | NM_173311 | 0.010 | +0.94 | 0.178 | -0.50 | 0.195 | +0.44 |
| | T-cell receptor beta chain | Tcrb | BC091428 | 0.010 | +0.77 | 0.222 | -0.36 | 0.152 | +0.40 |
| | unc-13 homolog D (C. elegans) | Unc13d | NM_138844 | 0.009 | +0.54 | 0.033 | -0.45 | 0.658 | +0.08 |
| | zeta-chain (TCR) associated protein kinase | Zap70 | NM_001012002 | 0.009 | +1.08 | 0.144 | -0.62 | 0.234 | +0.47 |
| Metabolism (carbohydrates) | glycosyltransferase 1 domain containing 1 | Glt1d1 | ENSRNOT00000064526 | 0.004 | -0.71 | 0.023 | +0.57 | 0.536 | -0.14 |
| | glucosamine-phosphate N-acetyltransferase 1 | Gnpnat1 | NM_001134757 | 0.010 | +0.60 | 0.062 | -0.45 | 0.488 | +0.15 |
| | heparanase | Hpse | NM_022605 | 0.005 | +0.82 | 0.036 | -0.63 | 0.484 | +0.19 |
| | phosphofructokinase, platelet | Pfkp | L25387 | 0.005 | +0.62 | 0.087 | -0.38 | 0.241 | +0.24 |
| Metabolism (vitamins and minerals) | iron responsive element binding protein 2 | Ireb2 | NM_022863 | 0.010 | +0.66 | 0.022 | -0.61 | 0.843 | +0.05 |
| | iron-sulfur cluster assembly 1 homolog (S. cerevisiae) | Isca1 | NM_181626 | 0.010 | -0.74 | 0.020 | +0.71 | 0.917 | -0.03 |
| | methylenetetrahydrofolate reductase (NAD(P)H) | Mthfr | ENSRNOT00000011384 | 0.010 | -0.57 | 0.086 | +0.39 | 0.389 | -0.18 |
| Metabolism (lipids) | acid phosphatase 6, lysophosphatidic | Acp6 | NM_001031645 | 0.002 | +0.75 | 0.101 | -0.40 | 0.122 | +0.35 |
| | choline phosphotransferase 1 | Chpt1 | NM_001007750 | 0.008 | +0.67 | 0.022 | -0.60 | 0.763 | +0.07 |
| | emopamil binding protein- | Ebpl | NM_001108381 | 0.003 | +0.77 | 0.106 | -0.42 | 0.150 | +0.35 |

| | | | | | | | | | |
|---|---|---|---|---|---|---|---|---|---|
| | like | | | | | | | | |
| | fatty acid amide hydrolase | Faah | NM_024132 | 0.007 | +0.79 | 0.187 | -0.39 | 0.137 | +0.41 |
| | low density lipoprotein receptor adaptor protein 1 | Ldlrap1 | NM_001109271 | 0.002 | +0.81 | 0.043 | -0.54 | 0.258 | +0.27 |
| | oxysterol binding protein-like 1A | Osbpl1a | NM_172023 | 0.002 | +0.75 | 0.037 | -0.52 | 0.304 | +0.23 |
| | 3-oxoacyl-ACP synthase, mitochondrial | Oxsm | NM_001100508 | 0.010 | +0.50 | 0.129 | -0.30 | 0.263 | +0.20 |
| | phosphatidylinositol glycan anchor biosynthesis, class L | Pigl | ENSRNOT00000004113 | 0.007 | +0.62 | 0.053 | -0.46 | 0.445 | +0.16 |
| | phospholipase A2, group XIIA | Pla2g12a | NM_001108565 | 0.004 | +0.77 | 0.042 | -0.54 | 0.358 | +0.22 |
| | ST8 alpha-N-acetyl-neuraminide alpha-2,8-sialyltransferase 1 | St8sia1 | NM_012813 | 0.008 | +0.91 | 0.053 | -0.68 | 0.472 | +0.23 |
| Metabolism (nucleotides) | adenylosuccinate lyase | Adsl | NM_001130503 | 0.010 | +0.48 | 0.090 | -0.33 | 0.368 | +0.16 |
| | phosphoribosylglycinamide formyltransferase | Gart | BC087644 | 0.010 | +0.48 | 0.067 | -0.35 | 0.467 | +0.13 |
| | 5'-nucleotidase, cytosolic III-like | Nt5c3l | NM_001007723 | 0.006 | +0.76 | 0.272 | -0.30 | 0.084 | +0.46 |
| | phosphoribosyl pyrophosphate synthetase-associated protein 1 | Prpsap1 | NM_022545 | 0.006 | +0.57 | 0.014 | -0.53 | 0.833 | +0.04 |
| Metabolism (proteins and polyamines) | anterior pharynx defective 1 homolog A (C. elegans) | Aph1a | NM_001014255 | 0.008 | -0.43 | 0.063 | +0.31 | 0.429 | -0.12 |
| | UDP-GlcNAc:betaGal beta-1,3-N-acetylglucosaminyltransferase 8 | B3gnt8 | NM_001107492 | 0.006 | -0.61 | 0.026 | +0.50 | 0.614 | -0.10 |
| | core 1 synthase, glycoprotein-N-acetylgalactosamine 3-beta-galactosyltransferase, 1 | C1galt1 | NM_022950 | 0.009 | +0.69 | 0.066 | -0.49 | 0.428 | +0.19 |
| | calreticulin | Calr | NM_022399 | 0.004 | -0.64 | 0.029 | +0.50 | 0.490 | -0.14 |
| | casein kinase 1, gamma 2 | Csnk1g2 | NM_023102 | 0.009 | +0.56 | 0.081 | -0.38 | 0.373 | +0.18 |
| | cathepsin W | Ctsw | NM_001024242 | 0.009 | +0.72 | 0.147 | -0.40 | 0.218 | +0.32 |
| | leucine carboxyl methyltransferase 1 | Lcmt1 | NM_199405 | 0.005 | +0.58 | 0.151 | -0.30 | 0.149 | +0.28 |
| | protein-L-isoaspartate (D-aspartate) O-methyltransferase domain containing 2 | Pcmtd2 | NM_001107810 | 0.000 | +0.72 | 0.158 | -0.27 | 0.018 | +0.45 |
| | protein arginine methyltransferase 3 | Prmt3 | NM_053557 | 0.003 | +0.74 | 0.052 | -0.49 | 0.281 | +0.25 |
| | proteinase 3 | Prtn3 | NM_001024264 | 0.006 | -0.69 | 0.023 | +0.59 | 0.657 | -0.10 |
| | ring finger protein 125 | Rnf125 | NM_001108424 | 0.009 | +0.94 | 0.150 | -0.52 | 0.216 | +0.42 |
| | serine (or cysteine) peptidase inhibitor, clade B, member 6b | Serpinb6b | NM_001012214 | 0.005 | +0.86 | 0.072 | -0.55 | 0.268 | +0.31 |
| | transglutaminase 1, K polypeptide | Tgm1 | NM_031659 | 0.003 | -0.87 | 0.432 | +0.23 | 0.024 | -0.64 |
| | transglutaminase 6 | Tgm6 | ENSRNOT00000009097 | 0.009 | -0.49 | 0.047 | +0.38 | 0.528 | -0.11 |
| | ubiquitin-like modifier activating enzyme 5 | Uba5 | NM_001009669 | 0.004 | +0.91 | 0.084 | -0.54 | 0.199 | +0.37 |
| | URI1, prefoldin-like chaperone | Uri1 | NM_001107507 | 0.002 | +0.95 | 0.014 | -0.78 | 0.532 | +0.17 |
| Metabolism (redox) | prolyl 4-hydroxylase, alpha polypeptide II | P4ha2 | NM_001108275 | 0.004 | -0.79 | 0.520 | +0.18 | 0.022 | -0.62 |
| | STEAP family member 4 | Steap4 | NM_001044265 | 0.006 | -0.51 | 0.118 | +0.29 | 0.214 | -0.22 |
| | thioredoxin interacting protein | Txnip | NM_001008767 | 0.001 | +0.91 | 0.098 | -0.46 | 0.079 | +0.46 |
| Neural signaling | 4-aminobutyrate aminotransferase | Abat | NM_031003 | 0.006 | +0.69 | 0.150 | -0.36 | 0.159 | +0.33 |
| | hippocalcin-like 1 | Hpcal1 | NM_017356 | 0.009 | +0.45 | 0.090 | -0.30 | 0.331 | +0.16 |
| | potassium voltage-gated channel, KQT-like subfamily, member 4 | Kcnq4 | AF249748 | 0.010 | -0.69 | 0.724 | +0.09 | 0.022 | -0.60 |
| | protein phosphatase 1, regulatory subunit 9A | Ppp1r9a | NM_053473 | 0.007 | -0.60 | 0.088 | +0.38 | 0.287 | -0.22 |
| Nervous system | adhesion molecule with Ig like domain 1 | Amigo1 | BC167749 | 0.005 | +0.87 | 0.062 | -0.59 | 0.336 | +0.28 |

| | | | | | | | | | |
|---|---|---|---|---|---|---|---|---|---|
| | churchill domain containing 1 | Churc1 | NM_001106741 | 0.009 | -0.52 | 0.306 | +0.20 | 0.099 | -0.31 |
| | membrane protein, palmitoylated 5 (MAGUK p55 subfamily member 5) | Mpp5 | NM_001108034 | 0.004 | +0.73 | 0.035 | -0.54 | 0.411 | +0.19 |
| | platelet-activating factor acetylhydrolase, isoform 1b, subunit 3 | Pafah1b3 | NM_053654 | 0.009 | +0.54 | 0.080 | -0.37 | 0.366 | +0.17 |
| | progesterone receptor membrane component 1 | Pgrmc1 | NM_021766 | 0.006 | +0.80 | 0.029 | -0.66 | 0.592 | +0.14 |
| | staufen, RNA binding protein, homolog 2 (Drosophila) | Stau2 | NM_001007149 | 0.004 | +0.77 | 0.019 | -0.65 | 0.608 | +0.12 |
| Others | angiomotin like 2 | Amotl2 | NM_031717 | 0.005 | -0.61 | 0.052 | +0.43 | 0.356 | -0.18 |
| | ADP-ribosylation factor-like 5A | Arl5a | ENSRNOT00000009181 | 0.005 | +0.55 | 0.103 | -0.32 | 0.202 | +0.23 |
| | glutathione S-transferase pi 1 | Gstp1 | NM_012577 | 0.007 | +0.59 | 0.044 | -0.45 | 0.477 | +0.14 |
| | mucin 5B, oligomeric mucus/gel-forming | Muc5b | ENSRNOT00000028967 | 0.009 | -0.93 | 0.024 | +0.84 | 0.794 | -0.09 |
| | nephrosis 1, congenital, Finnish type | Nphs1 | NM_022628 | 0.006 | -0.68 | 0.202 | +0.31 | 0.118 | -0.36 |
| | SPO11 meiotic protein covalently bound to DSB homolog (S. cerevisiae) | Spo11 | NM_001108964 | 0.006 | +0.62 | 0.101 | -0.37 | 0.234 | +0.25 |
| | zona pellucida binding protein 2 | Zpbp2 | NM_001007011 | 0.005 | +0.60 | 0.079 | -0.38 | 0.253 | +0.22 |
| Sensory perception | cyclic nucleotide gated channel alpha 1 | Cnga1 | NM_053497 | 0.006 | +0.72 | 0.138 | -0.39 | 0.168 | +0.33 |
| | olfactory receptor 107 | Olr107 | NM_001000148 | 0.006 | -0.64 | 0.037 | +0.50 | 0.490 | -0.15 |
| | olfactory receptor 1075 | Olr1075 | NM_001000421 | 0.001 | -0.95 | 0.015 | +0.72 | 0.366 | -0.23 |
| | olfactory receptor 1394 | Olr1394 | NM_001001091 | 0.002 | +0.72 | 0.027 | -0.53 | 0.373 | +0.19 |
| | olfactory receptor 1481 | Olr1481 | NM_001000527 | 0.008 | -0.43 | 0.158 | +0.23 | 0.194 | -0.20 |
| | olfactory receptor 1500 | Olr1500 | NM_001000942 | 0.001 | +0.71 | 0.347 | -0.19 | 0.012 | +0.51 |
| | olfactory receptor 1602 | Olr1602 | NM_001000909 | 0.004 | -0.71 | 0.274 | +0.26 | 0.055 | -0.45 |
| | olfactory receptor 239 | Olr239 | NM_001000211 | 0.005 | -0.52 | 0.557 | +0.11 | 0.020 | -0.42 |
| | olfactory receptor 439 | Olr439 | NM_001000281 | 0.009 | +0.74 | 0.028 | -0.64 | 0.722 | +0.09 |
| | olfactory receptor 500 | Olr500 | NM_001000680 | 0.006 | +0.58 | 0.493 | -0.14 | 0.032 | +0.44 |
| Signaling | axin 2 | Axin2 | NM_024355 | 0.009 | +0.65 | 0.143 | -0.37 | 0.234 | +0.28 |
| | calcium/calmodulin-dependent protein kinase IV | Camk4 | NM_012727 | 0.009 | +0.81 | 0.049 | -0.63 | 0.539 | +0.18 |
| | CD38 molecule | Cd38 | NM_013127 | 0.006 | +0.81 | 0.072 | -0.54 | 0.314 | +0.27 |
| | COMM domain containing 7 | Commd7 | NM_001030029 | 0.008 | +0.63 | 0.149 | -0.34 | 0.192 | +0.29 |
| | diacylglycerol kinase, alpha | Dgka | NM_080787 | 0.010 | +0.92 | 0.168 | -0.49 | 0.205 | +0.42 |
| | GDP dissociation inhibitor 1 | Gdi1 | NM_017088 | 0.010 | +0.47 | 0.035 | -0.40 | 0.689 | +0.07 |
| | interleukin 21 receptor | Il21r | NM_001012469 | 0.010 | +0.78 | 0.170 | -0.42 | 0.208 | +0.36 |
| | interleukin 7 receptor | Il7r | NM_001106418 | 0.008 | +0.84 | 0.039 | -0.69 | 0.594 | +0.16 |
| | myelin protein zero-like 1 | Mpzl1 | NM_001007728 | 0.008 | -0.53 | 0.121 | +0.32 | 0.249 | -0.22 |
| | nemo like kinase | Nlk | NM_001191924 | 0.008 | +0.57 | 0.017 | -0.54 | 0.884 | +0.03 |
| | natriuretic peptide receptor A/guanylate cyclase A (atrionatriuretic peptide receptor A) | Npr1 | NM_012613 | 0.005 | -0.40 | 0.735 | +0.05 | 0.012 | -0.35 |
| | progestin and adipoQ receptor family member V | Paqr5 | NM_001014092 | 0.007 | +0.97 | 0.154 | -0.51 | 0.177 | +0.46 |
| | protein kinase (cAMP-dependent, catalytic) inhibitor alpha | Pkia | FQ211701 | 0.003 | +0.92 | 0.046 | -0.62 | 0.286 | +0.30 |
| | phospholipase C, gamma 1 | Plcg1 | NM_013187 | 0.008 | +0.57 | 0.186 | -0.29 | 0.159 | +0.29 |
| | protein kinase C, theta | Prkcq | ENSRNOT00000025901 | 0.007 | +0.95 | 0.097 | -0.59 | 0.274 | +0.36 |
| | RAS p21 protein activator 4 | Rasa4 | XM_002724808 | 0.006 | +0.53 | 0.241 | -0.23 | 0.098 | +0.30 |
| | ret proto-oncogene | Ret | NM_001110099 | 0.008 | +0.90 | 0.168 | -0.47 | 0.173 | +0.43 |
| | regulator of G-protein signaling 1 | Rgs1 | NM_019336 | 0.007 | +1.73 | 0.076 | -1.16 | 0.333 | +0.58 |
| | signal transducer and activator of transcription 4 | Stat4 | NM_001012226 | 0.007 | +0.80 | 0.225 | -0.36 | 0.113 | +0.44 |
| | serine/threonine kinase 4 | Stk4 | NM_001107800 | 0.002 | +0.74 | 0.023 | -0.53 | 0.324 | +0.21 |
| | tetraspanin 6 | Tspan6 | NM_001100672 | 0.008 | +0.53 | 0.101 | -0.33 | 0.279 | +0.20 |
| | tyrosine 3-monooxygenase/tryptophan 5-monooxygenase activation protein, zeta polypeptide | Ywhaz | NM_013011 | 0.008 | +0.62 | 0.019 | -0.57 | 0.822 | +0.05 |

| | | | | | | | | | |
|---|---|---|---|---|---|---|---|---|---|
| | Rho guanine nucleotide exchange factor (GEF) 1 | Arhgef1 | NM_021694 | 0.006 | +0.63 | 0.103 | -0.38 | 0.243 | +0.25 |
| Transcription / translation machinery | cirrhosis, autosomal recessive 1A (cirhin) | Cirh1a | NM_001009640 | 0.002 | +0.67 | 0.066 | -0.40 | 0.183 | +0.27 |
| | CREB regulated transcription coactivator 1 | Crtc1 | NM_001047115 | 0.000 | -0.89 | 0.121 | +0.36 | 0.019 | -0.53 |
| | DEAD (Asp-Glu-Ala-Asp) box polypeptide 50 | Ddx50 | NM_001013198 | 0.005 | +0.61 | 0.049 | -0.43 | 0.367 | +0.18 |
| | DEAH (Asp-Glu-Ala-His) box polypeptide 32 | Dhx32 | NM_001130039 | 0.005 | +0.49 | 0.016 | -0.44 | 0.714 | +0.06 |
| | DMRT-like family C1a | Dmrtc1a | NM_001025288 | 0.005 | -0.75 | 0.037 | +0.58 | 0.481 | -0.17 |
| | eukaryotic translation elongation factor 2 | Eef2 | NM_017245 | 0.006 | +0.58 | 0.024 | -0.49 | 0.633 | +0.09 |
| | eukaryotic translation initiation factor 3, subunit E | Eif3e | NM_001011990 | 0.010 | +0.56 | 0.044 | -0.45 | 0.606 | +0.10 |
| | FtsJ methyltransferase domain containing 1 | Ftsjd1 | NM_001106186 | 0.007 | +0.54 | 0.025 | -0.46 | 0.666 | +0.08 |
| | GAR1 ribonucleoprotein homolog (yeast) | Gar1 | NM_001024306 | 0.006 | +0.78 | 0.021 | -0.69 | 0.716 | +0.09 |
| | Meis homeobox 3 | Meis3 | NM_001108472 | 0.003 | +0.64 | 0.068 | -0.39 | 0.198 | +0.25 |
| | nuclear receptor co-repressor 1 | Ncor1 | XM_001077495 | 0.005 | +0.54 | 0.024 | -0.45 | 0.629 | +0.09 |
| | NIN1/RPN12 binding protein 1 homolog (S. cerevisiae) | Nob1 | NM_199086 | 0.009 | +0.55 | 0.048 | -0.43 | 0.526 | +0.12 |
| | nucleolar complex associated 2 homolog (S. cerevisiae) | Noc2l | NM_001033897 | 0.008 | +0.61 | 0.033 | -0.52 | 0.646 | +0.10 |
| | nucleolar protein 9 | Nol9 | ENSRNOT00000013535 | 0.010 | +0.54 | 0.064 | -0.40 | 0.478 | +0.14 |
| | similar to phosphoseryl-tRNA kinase | Pstk | ENSRNOT00000027967 | 0.009 | +0.59 | 0.083 | -0.40 | 0.359 | +0.19 |
| | RNA binding motif (RNP1, RRM) protein 3 | Rbm3 | NM_053696 | 0.008 | +0.46 | 0.173 | -0.24 | 0.170 | +0.22 |
| | similar to 40S ribosomal protein S19 | RGD1559724 | ENSRNOT00000047882 | 0.010 | +0.75 | 0.077 | -0.53 | 0.406 | +0.22 |
| | similar to phosphoseryl-tRNA kinase | RGD1564300 | BC168162 | 0.010 | +0.70 | 0.052 | -0.54 | 0.533 | +0.16 |
| | ribosomal protein S11 | Rps11 | NM_031110 | 0.006 | +0.75 | 0.033 | -0.61 | 0.569 | +0.14 |
| | ribosomal protein S19 | Rps19 | NM_001037346 | 0.003 | +0.78 | 0.035 | -0.57 | 0.386 | +0.21 |
| | SATB homeobox 1 | Satb1 | NM_001012129 | 0.004 | +0.90 | 0.112 | -0.50 | 0.176 | +0.40 |
| | T-box 3 | Tbx3 | NM_181638 | 0.010 | -0.52 | 0.107 | +0.33 | 0.309 | -0.19 |
| | transcription factor 12 | Tcf12 | NM_013176 | 0.008 | +0.80 | 0.031 | -0.67 | 0.649 | +0.13 |
| | tRNA methyltransferase 11 homolog (S. cerevisiae) | Trmt11 | ENSRNOT00000019436 | 0.006 | +0.55 | 0.046 | -0.41 | 0.436 | +0.14 |
| | TSR2, 20S rRNA accumulation, homolog (S. cerevisiae) | Tsr2 | NM_001115027 | 0.009 | +0.61 | 0.078 | -0.42 | 0.380 | +0.19 |
| | UPF3 regulator of nonsense transcripts homolog B (yeast) | Upf3b | NM_001135873 | 0.009 | +0.46 | 0.085 | -0.31 | 0.354 | +0.15 |
| | WD repeat domain 77 | Wdr77 | NM_001008771 | 0.006 | +0.63 | 0.119 | -0.36 | 0.199 | +0.27 |
| | zinc finger CCCH-type containing 15 | Zc3h15 | NM_001010963 | 0.002 | +0.58 | 0.059 | -0.35 | 0.179 | +0.23 |
| | zinc finger protein 503 | Zfp503 | NM_001107250 | 0.002 | -0.52 | 0.031 | +0.37 | 0.316 | -0.15 |
| | zinc finger protein 638 | Zfp638 | NM_001107868 | 0.006 | +0.45 | 0.019 | -0.40 | 0.722 | +0.05 |
| | zinc finger protein 709 | Zfp709 | NM_153731 | 0.007 | +0.51 | 0.119 | -0.30 | 0.234 | +0.21 |
| | zinc finger, HIT-type containing 6 | Znhit6 | NM_001106203 | 0.010 | +0.69 | 0.070 | -0.50 | 0.432 | +0.20 |
| | B-cell receptor-associated protein 29 | Bcap29 | NM_001006980 | 0.001 | +0.76 | 0.057 | -0.45 | 0.144 | +0.32 |
| Transport | cytohesin 1 | Cyth1 | NM_053910 | 0.005 | +0.70 | 0.112 | -0.39 | 0.186 | +0.30 |
| | potassium large conductance calcium-activated channel, subfamily M, beta member 4 | Kcnmb4 | NM_023960 | 0.007 | +0.81 | 0.146 | -0.44 | 0.190 | +0.37 |
| | NIPA-like domain containing 1 | Nipal1 | NM_001106003 | 0.007 | +0.52 | 0.107 | -0.31 | 0.254 | +0.20 |
| | phosphofurin acidic cluster sorting protein 1 | Pacs1 | NM_134406 | 0.004 | +0.60 | 0.105 | -0.34 | 0.175 | +0.26 |
| | sodium channel, nonvoltage-gated 1, beta | Scnn1b | NM_012648 | 0.009 | +0.49 | 0.131 | -0.28 | 0.242 | +0.20 |
| | solute carrier family 12 (potassium/chloride transporters), member 7 | Slc12a7 | NM_001013144 | 0.010 | +0.56 | 0.096 | -0.37 | 0.349 | +0.19 |

| | | | | | | | | |
|---|---|---|---|---|---|---|---|---|
| solute carrier family 16, member 8 (monocarboxylic acid transporter 3) | Slc16a8 | NM_031744 | 0.005 | -0.51 | 0.081 | +0.32 | 0.267 | -0.19 |
| solute carrier family 18 (vesicular monoamine), member 2 | Slc18a2 | NM_013031 | 0.010 | +0.72 | 0.122 | -0.44 | 0.275 | +0.28 |
| solute carrier family 34 (sodium phosphate), member 3 | Slc34a3 | NM_139338 | 0.007 | +0.59 | 0.352 | -0.20 | 0.062 | +0.39 |
| solute carrier family 9 (sodium/hydrogen exchanger), isoform 9 | Slc9a9 | XM_001064905 | 0.010 | +0.56 | 0.100 | -0.36 | 0.331 | +0.20 |
| TSC22 domain family, member 3 | Tsc22d3 | NM_031345 | 0.002 | +0.86 | 0.138 | -0.39 | 0.066 | +0.46 |
| VAMP (vesicle-associated membrane protein)-associated protein B and C | Vapb | NM_021847 | 0.004 | +0.51 | 0.011 | -0.47 | 0.805 | +0.04 |

Controls: the offspring of rats with free access to standard chow diet; CR: the offspring of 20% calorie restricted dams during the first 12 days of pregnancy; CR-Leptin: CR rats daily supplemented with physiological doses of leptin throughout lactation. *p*-values (*P)* of microarray data (limma *t*-test) and fold change (FC) values (calculated as the difference between Log2 means) of CR *vs* Controls, CR-Leptin vs CR and CR-Leptin *vs* Controls comparisons are indicated; +, indicates upregulation; –, downregulation. Threshold of significance was set at $p \leq 0.010$.

**Blood cell transcriptomic-based early biomarkers of adverse programming effects of gestational calorie restriction and their reversibility by leptin supplementation**

Jadwiga Konieczna, Juana Sánchez, Mariona Palou, Catalina Picó*, Andreu Palou

Supplementary table 2. Function of selected genes from the microarray analysis of PBMC samples of male rats at the age of 25 days which expression was affected by gestational calorie restriction and became totally reverted by oral leptin supplementation throughout lactation.

| Gene symbol | Gene name | Protein codified | Function |
|---|---|---|---|
| *Crmp1* | Collapsin response mediator protein 1 | CRMP1 | Member of the Collapsin response mediator protein (CRMP) family of proteins highly expressed in developing and adult nervous systems and implicated in axon guidance and outgrowth [1]. *Crmp1* has been characterized as a potential invasion-suppressor gene [2]. |
| *Diexf* | Digestive Organ Expansion Factor Homolog (Zebrafish) | DIEXF | In zebrafish, it acts as a pan-endoderm factor to coordinate the expansion growth of the entire digestive system. A loss-of-function mutation in Zebrafish of *Diexf* gene results in compromised organ growth [3]. |
| *Gla* | Galactosidase, alpha | GLA | Homodimeric glycoprotein that hydrolyses the terminal alpha-galactosyl moieties from glycolipids and glycoproteins. Defects in GLA activity, which is characteristic of the Fabry disease, leads to the systemic accumulation of neutral glycosphingolipids with terminal alpha-galactosyl moieties [4]. |
| *Gls* | Glutaminase | GLS | It catalyzes the hydrolysis of glutamine to glutamate and ammonia. This protein is primarily expressed in the brain and kidney and plays an essential role in generating energy for metabolism, synthesizing the brain neurotransmitter glutamate and maintaining acid-base balance in the kidney [5]. |
| *Lrp11* | Low density lipoprotein receptor-related protein 11 | LRP11 | Member of the LDL receptor family, a class of structurally closely related cell surface receptors fulfilling diverse functions in different tissues, which has been recently positioned as one of the key players in Alzheimer disease research [6]. |
| *Paox* | Polyamine oxidase (exo-N4-amino) | PAO | Flavoenzyme that catalyzes the oxidation of N(1)-acetylspermine to spermidine and hence is involved in the polyamine back-conversion, thus playing an important role in the regulation of polyamine intracellular concentration. However, PAO activity results in production of ammonia, the corresponding amino aldehydes, and hydrogen peroxide. Malondialdehyde (MDA) and acrolein, potentially toxic agents, which induce oxidative stress in mammalian cells, are spontaneously formed from aminoaldehydes. Thus, resulting products of PAO activity have the potential to produce disease states. Increased PAO activity has been found in type 1 diabetic children, associated to increased blood HbA(1C) and MDA levels, demonstrating that increased plasma PAO |

| | | | |
|---|---|---|---|
| | | | activity may participate in these circumstances [7]. Activation of PAO has also been associated to macrophage apoptosis due to hydrogen peroxide release and mitochondrial membrane depolarization, contributing to deficiencies in host defence in diseases such as H. pylori infection [8]. |
| *Rnf10* | Ring finger protein 10 | RNF10 | The protein contains a ring finger motif, which is known to be involved in protein-protein interactions. The specific function of this protein has not yet been determined, but recent findings pinpoint its critical role in myelin formation and neuronal differentiation [9,10]. |
| *Selenbp1* | Selenium binding protein 1 | SELENBP1 | Member of selenoproteins family. These proteins bind selenium covalently and mediate the intracellular transport of selenium [11]. A deficiency of dietary selenium is associated with an increased incidence of epithelial cancers including lung, liver, colorectal, and prostate cancer [12]. Selenium exerts its anticarcinogenic effects mainly through selenoproteins, and expression of SELENBP1 has been found to be reduced markedly in multiple epithelial cancers compared with their corresponding normal tissues. The exact function of SELENBP1 is not known, although, a possible link to malignancies associated with selenium deficiencies has been suggested [13,14]. |
| *Slc7a5* | Solute carrier family 7 (amino acid transporter light chain, L system), member 5 | SLC7A5, LAT1 | It is involved in the sodium-independent cellular transport of amino acids with large neutral amino acid side chains [15]. Increased expression levels of *Slc7a5* gene were positively correlated with increased biological aggressiveness and higher mortality in a range of human cancers [16]. Expression levels of this gene may be nutritionally modulated, since decreased expression levels were described in brain tissues of pigs fed low dietary protein [17]. |
| *Tmsb4x* | Thymosin beta 4, X-linked | Tß4 | The major actin-sequestering protein in all eukaryotic cells and a potent regulator of actin polymerization in mammals [18]. Numerous studies have identified a range of functions and activities for Tß4 important for wound healing and repair and tissue regeneration [18]. Tß4 also appears to be involved in the regulation of the development and regeneration of the nervous system, as a novel neurotrophic signal [19]. It is highly expressed in most neural cell types of the developing brain, and has also been found to be abundant in the injured/regenerating axons, suggesting that the upregulation of Tß4 is related to the axonal sprouting and neuronal regeneration. On the other hand, up-regulation of *Tmsb4x* gene has been discovered in a wide variety of human carcinomas and has been proposed as a key event in the acquisition of growth advantages as well as invasive phenotypes in human colorectal carcinomas [20]. |
| *Ubash3b* | Ubiquitin associated and SH3 domain containing, B | UBASH3B, STS-1 | The protein exhibits tyrosine phosphatase activity toward several substrates. This protein contains an ubiquitin associated domain at the N-terminus. Proteins containing ubiquitin-binding domains (UBDs) interact with ubiquitinated targets and regulate diverse biological processes, including endocytosis, signal transduction, transcription and DNA repair [21]. Overexpression of this gene has been identified in aggressive cancers like triple-negative breast cancer, and promotes invasion and metastasis [22]. |

**Reference list**


1. Quinn, C. C., Gray, G. E. & Hockfield, S. A family of proteins implicated in axon guidance and outgrowth. *J Neurobiol.* **41**, 158-164 (1999).
2. Shih, J. Y. *et al.* Collapsin response mediator protein-1 and the invasion and metastasis of cancer cells. *J Natl Cancer Inst.* **93**, 1392-1400 (2001).
3. Chen, J. *et al.* Loss of function of def selectively up-regulates Delta113p53 expression to arrest expansion growth of digestive organs in zebrafish. *Genes Dev* **19**, 2900-2911 (2005).
4. Mehta, A. *et al.* Fabry disease: a review of current management strategies. *QJM.* **103**, 641-659 (2010).
5. Marquez, J., de la Oliva, A. R., Mates, J. M., Segura, J. A. & Alonso, F. J. Glutaminase: a multifaceted protein not only involved in generating glutamate. *Neurochem. Int* **48**, 465-471 (2006).
6. Jaeger, S. & Pietrzik, C. U. Functional role of lipoprotein receptors in Alzheimer's disease. *Curr Alzheimer Res* **5**, 15-25 (2008).
7. Bjelakovic, G. *et al.* Does polyamine oxidase activity influence the oxidative metabolism of children who suffer of diabetes mellitus? *Mol Cell Biochem* **341**, 79-85 (2010).
8. Chaturvedi, R. *et al.* Induction of polyamine oxidase 1 by Helicobacter pylori causes macrophage apoptosis by hydrogen peroxide release and mitochondrial membrane depolarization. *J Biol Chem* **279**, 40161-40173 (2004).
9. Hoshikawa, S., Ogata, T., Fujiwara, S., Nakamura, K. & Tanaka, S. A novel function of RING finger protein 10 in transcriptional regulation of the myelin-associated glycoprotein gene and myelin formation in Schwann cells. *PLoS ONE* **3**, e3464 (2008).
10. Malik, Y. S., Sheikh, M. A., Lai, M., Cao, R. & Zhu, X. RING finger protein 10 regulates retinoic acid-induced neuronal differentiation and the cell cycle exit of P19 embryonic carcinoma cells. *J Cell Biochem* **114**, 2007-2015 (2013).
11. Behne, D. & Kyriakopoulos, A. Mammalian selenium-containing proteins. *Annu Rev Nutr* **21**, 453-473 (2001).
12. Virtamo, J. *et al.* Serum selenium and risk of cancer. A prospective follow-up of nine years. *Cancer* **60**, 145-148 (1987).
13. Chen, G. *et al.* Reduced selenium-binding protein 1 expression is associated with poor outcome in lung adenocarcinomas. *J Pathol.* **202**, 321-329 (2004).
14. Zeng, G. Q. *et al.* The function and significance of SELENBP1 downregulation in human bronchial epithelial carcinogenic process. *PLoS ONE* **8**, e71865 (2013).
15. Kanai, Y. *et al.* Expression cloning and characterization of a transporter for large neutral amino acids activated by the heavy chain of 4F2 antigen (CD98). *J Biol Chem* **273**, 23629-23632 (1998).
16. Fuchs, B. C. & Bode, B. P. Amino acid transporters ASCT2 and LAT1 in cancer: partners in crime? *Semin. Cancer Biol* **15**, 254-266 (2005).
17. Wu, X. *et al.* Dietary protein, energy and arginine affect LAT1 expression in forebrain white matter differently. *Animal.* **4**, 1518-1521 (2010).
18. Crockford, D., Turjman, N., Allan, C. & Angel, J. Thymosin beta4: structure, function, and biological properties supporting current and future clinical applications. *Ann N Y Acad Sci* **1194**, 179-189 (2010).
19. Sun, W. & Kim, H. Neurotrophic roles of the beta-thymosins in the development and regeneration of the nervous system. *Ann N Y Acad Sci* **1112**, 210-218 (2007).
20. Wang, W. S. *et al.* Overexpression of the thymosin beta-4 gene is associated with increased invasion of SW480 colon carcinoma cells and the distant metastasis of human colorectal carcinoma. *Oncogene* **23**, 6666-6671 (2004).
21. Hoeller, D. *et al.* Regulation of ubiquitin-binding proteins by monoubiquitination. *Nat Cell Biol* **8**, 163-169 (2006).
22. Lee, S. T. *et al.* Protein tyrosine phosphatase UBASH3B is overexpressed in triple-negative breast cancer and promotes invasion and metastasis. *Proc Natl Acad Sci U. S. A* **110**, 11121-11126 (2013).
